# Encryption chain based on measurement result and its applications on semi-quantum key distribution protocol

**DOI:** 10.1038/s41598-022-23135-7

**Published:** 2022-11-01

**Authors:** Chun-Wei Yang

**Affiliations:** Master Program for Digital Health Innovation, College of Humanities and Sciences, China Medical University, No. 100, Sec. 1, Jingmao Rd., Beitun Dist., Taichung, 406040 Taiwan, ROC

**Keywords:** Quantum physics, Quantum information

## Abstract

This study proposes a new encoding method, also known as an encryption chain based on the measurement result. Then, using the encryption chain to propose a unitary-operation-based semi-quantum key distribution protocol (SQKD) protocol. In the existing SQKD protocols, semi-quantum environments adopt a round-trip transmission strategy. In round-trip transmission, the classical participant must resend the received photons to the quantum participant after implementing local operations. Therefore, round-trip transmissions are vulnerable to Trojan horse attacks. Hence, the classical participant must be equipped with a photon number splitter and an optical wavelength filter device against Trojan horse attacks. This is illogical for semi-quantum environments because the burden on the classical participant is significantly increased as it involves the prevention of Trojan horse attacks. The proposed SQKD protocol is congenitally immune to Trojan horse attacks and involves no extra hardware because it is designed based on a one-way transmission as opposed to a round-trip transmission. When compared to the existing SQKD protocols, the proposed SQKD protocol provides the best qubit efficiency, and classical participants only require two quantum capabilities, which enhance its practicability. Moreover, the proposed SQKD protocol is free from collective attacks, Trojan horse attacks, and intercept-resend attacks. Thus, the proposed scheme is more efficient and practical than the existing SQKD protocols.

## Introduction

With the development of information technology, breakthroughs and innovations in the internet of things (IoT), cloud computing, big data, and artificial intelligence (AI) technologies, AI and IoT techniques are used to help solve problems are becoming increasingly popular, especially in the medical field^1–5^. To ensure the data security of these applications, most of them use encryption techniques to protect data security. However, to securely create the secret keys required for encryption, many mainstream applications use public-key cryptographic system to distribute secret keys. In 1994, Shor proposed a quantum algorithm^6^ that can break the RSA encryption system in a polynomial time. Therefore, the security framework of the RSA encryption system, which is based on the mathematical difficulty of prime factorization, cannot be guaranteed in the environment of quantum computers. This groundbreaking research result also drives the research energy of quantum cryptography. Therefore, how to design cryptographic techniques that can resist quantum computer attacks has become an important issue in cryptographic research.

Since the rapid development of quantum communication, quantum key distribution (QKD) protocol has become one of the most critical research areas in quantum cryptography. The main principle of the QKD protocol involves distributing a secret key to a receiver via the transmission of qubits. In 1984, Bennett and Brassard^7^ developed the first QKD scheme, termed the BB84 protocol, based on the properties of quantum mechanics. In 1992, Bennett et al.^8^ put forward a QKD protocol based on the Bell states. In 2002, Long and Liu^9^ proposed the QKD protocol by means of a two-step communication strategy. In 2003, Deng et al.^10^ also developed a two-step quantum secure direct communication (QSDC) protocol based on Long and Liu’s concept. Unlike the QKD protocol, the QSDC protocol allows two participants to transmit information directly over a quantum channel without sharing any secrets in advance. Subsequently, numerous QKD protocols^11–23^ and QSDC protocols^24–28^ have been proposed using single photons or entangled states. Although QKD protocols provide unconditional security^29–32^, it must be assumed that the sender and the receiver possess unlimited quantum capabilities, including generating single photons or entangled states, measuring qubits with any basis, and storing qubits in a quantum register. However, most quantum capabilities are difficult to implement, and the devices are expensive. Some researchers focused on designing semi-quantum key distribution (SQKD) protocols that can lead to a more practical QKD protocol.

Boyer et al.^33^ developed the first SQKD protocol in 2007. In 2009, Boyer et al.^34^ developed two SQKD schemes and defined two semi-quantum environments: the randomization-based environment and measure–resend environment. Based on Boyer et al.’s definition, the term “semi-quantum” implies that the sender, Alice, is a powerful quantum participant, whereas the receiver, Bob, solely possesses classical capabilities. Quantum participants can perform actions such as quantum generation, measurement, and storage. However, the receiver is restricted to implementing the following operations: (1) perform Z-basis $\left\{\left|0\rangle , \right.\left|1\rangle \right.\right\}$ measurement; (2) generate photons using Z-basis; (3) reflect the photons without any disturbance; and (4) reorder photons using different delay lines. Regarding the limitation of quantum capabilities, the randomization-based SQKD protocol assumes that the receiver possesses three types of quantum capabilities: (1) perform Z-basis measurement; (2) reflect the photons without any disturbance; and (3) reorder photons using different delay lines. The measure–resend SQKD protocol assumes that the receiver possesses three types of quantum capabilities: (1) perform Z-basis measurement; (2) generate photons using Z-basis; and (3) reflect the photons without any disturbance. After the semi-quantum concept was presented, various SQKD protocols^35–47^ were proposed for different security scenarios.

Based on a different perspective, Lo et al.^48^ developed the first measurement device-independent (MDI) QKD protocol in 2012. MDI-QKD protocols can be free from various eavesdropping attacks on qubit detectors and have been experimentally implemented^49–52^. In MDI-QKD protocols, the communicators send qubits to a third party (TP), which conducts a Bell-state analysis (BSA). Hence, the TP can be untrusted. That is, TP can be completely controlled by an eavesdropper.

Similarly, Zou et al.^53^ further restricted the abilities of classical participants. In 2015, Zou et al.^53^ proposed an SQKD protocol without invoking the measurement capability of a classical participant and proved it as robust with respect to quantum joint attacks. Regarding the limitation of quantum capabilities, the measurement-free SQKD protocol assumes that the receiver possesses three types of quantum capabilities: (1) generate photons using Z-basis; (2) reflect the photons without any disturbance; and (3) reorder photons using different delay lines. In 2018, Liu and Hwang^54^ designed a mediated SQKD (MSQKD) protocol using a measurement-free environment, where the TP should also be equipped with an entangled state generator, an entangled state measurement device, and a quantum register or a quantum delay line.

In contrast to the aforementioned SQKD or MSQKD protocols, Tsai et al.^55,56^ proposed lightweight MSQKD protocols, in which the classical participants only possess the capabilities of (1) performing Z-basis measurement and (2) performing the unitary operation. Moreover, Tsai et al.’s lightweight MSQKD protocol^55,56^ can reduce the quantum capabilities of the TP. That is, the TP has only two quantum capabilities: (1) generate photons using Z-basis and (2) perform the unitary operation. This implies that TP and communicators are classical in Tsai et al.’s lightweight MSQKD protocol^55,56^. In other words, to implement a semi-quantum cryptographic protocol, the classical participant does not require a quantum-generating device. With respect to the limitation of quantum capabilities, the semi-quantum cryptographic protocols based on a unitary-operation-based environment assume that the receiver possesses two types of quantum capabilities: (1) performing Z-basis measurement and (2) performing the unitary operation. In 2020, Tsai and Yang^57^ designed a lightweight authenticated SQKD (ASQKD) protocol using the Bell states. When compared to existing ASQKD protocols^58–63^, Tsai and Yang’s scheme only requires the classical participant to possess only two quantum capabilities. Thus, Tsai and Yang’s scheme is less demanding than existing ASQKD protocols in terms of practical implementation.

Based on the qubit’s transmission strategy, the quantum cryptographic protocols presented, to date, can generally be classified into three types: quantum-relay transmission, round-trip transmission, and one-way transmission. Specifically, these semi-quantum environments (i.e., randomization-based, measure-resend, and measurement-free) adopt a round-trip transmission strategy. In round-trip transmission, the classical participant must send back the received qubits to the quantum participant after performing measurements or operations. That is, the qubits are received and sent to the other participants. Hence, round-trip transmissions can suffer from Trojan horse attacks^64–66^. To address the problem of Trojan horse attacks, the classical participant must be equipped with a photon number splitter^67^ and an optical wavelength filter device^68^ against Trojan horse attacks. This is illogical for semi-quantum environments because the burden on the classical participant is significantly increased by the threat of Trojan horse attacks. Thus, these semi-quantum cryptographic protocols introduce high overheads, which significantly reduce communication efficiency.

In this work, an SQKD protocol is designed based on Bell state $\left|{\Phi }^{+}\rangle \right.=\frac{1}{\sqrt{2}}(\left|00\rangle \right.+\left|11\rangle \right.)$ and one-way transmission. The designed SQKD protocol is developed based on one-way transmission as opposed to round-trip transmission, which enhances its practicability. Specifically, the qubits are directly distributed by the quantum participant to the classical participant via a single path. In addition, this work proposed a new coding function for a unitary-operation-based environment, i.e., the quantum communicator and classical communicator decide to perform the identity operation or Hadamard operation on one of the two-particle quantum entanglement $\left|{\Phi }^{+}\rangle \right.$ based on the previous measurement result. For example, if the previous measurement result is $\left|0\right.\rangle (|1\rangle )$, then the quantum communicator and classical communicator perform the identity operation (the Hadamard operation) on the qubit and measure it using a Z-basis. By using the measurement property of Bell states and Hadamard operation, when the quantum communicator and classical communicator perform the Hadamard operation on first and second qubits from each $\left|{\Phi }^{+}\rangle \right.$, they can obtain the same measurement results using a Z-basis measurement. Based on the measurement results, the quantum and classical communicators can share a secret key. Therefore, the proposed SQKD protocol exhibits the following advantages over existing SQKD protocols.

1. It is simple and efficient because the classical participant only performs Z-basis measurement and Hadamard operation.
2. It is secure with respect to Trojan-horse attack because one-way transmission is adopted.
3. It is immune to various individual eavesdropping attacks.

The remainder of this paper is organized as follows. In “Encryption chain based on the measurement result”, a new coding function is presented based on Bell states and Hadamard operations. In “Proposed unitary-operation-based SQKD protocol”, a unitary-operation-based SQKD protocol is described. In “Security analysis”, an analysis of the security of the proposed SQKD protocol is presented. In “Efficiency analysis”, an analysis of the efficiency of the proposed scheme is presented. Finally, the conclusions of the study are stated in “Conclusion”.

## Encryption chain based on the measurement result

In this section, the relationship between Bell states and Hadamard operations is first introduced. In “Encryption chain for the encoding function” and “Encryption chain for the decoding function”, based on the measurement result, an encryption chain for new encoding and decoding functions is proposed. The coding function is useful for constructing a unitary-operation-based SQKD protocol for participants with different abilities.

### Relationship between Bell states and Hadamard operations

The Bell state, as known as the EPR pair, is a two-particle quantum-entangled state. Bell states have the four orthogonal maximal states and can be represented as follows:

1 $$\begin{gathered} \left| {\Phi^{ + } } \right\rangle = \frac{1}{\sqrt 2 }\left( {\left| {00} \right\rangle + \left| {11} \right\rangle } \right) \hfill \\ \left| {\Phi^{ - } } \right\rangle = \frac{1}{\sqrt 2 }\left( {\left| {00} \right\rangle - \left| {11} \right\rangle } \right) \hfill \\ \left| {\Psi^{ + } } \right\rangle = \frac{1}{\sqrt 2 }\left( {\left| {01} \right\rangle + \left| {10} \right\rangle } \right) \hfill \\ \left| {\Psi^{ - } } \right\rangle = \frac{1}{\sqrt 2 }\left( {\left| {01} \right\rangle - \left| {10} \right\rangle } \right) \hfill \\ \end{gathered}$$

Regarding the limitation of quantum capabilities, the unitary-operation-based environment^35–37^ assumes that the receiver possesses two types of quantum capabilities: (1) performing Z-basis measurement and (2) performing identity operator *I* or Hadamard operator *H*, where* I* and *H* are defined as follows:

2 $$I = \left| 0 \right\rangle \left\langle 0 \right| + \left| 1 \right\rangle \left\langle 1 \right| = \left[ {\begin{array}{*{20}c} 1 & 0 \\ 0 & 1 \\ \end{array} } \right]$$

3 $$H = \frac{1}{\sqrt 2 }\left( {\left| 0 \right\rangle \left\langle 0 \right| + \left| 1 \right\rangle \left\langle 0 \right| + \left| 0 \right\rangle \left\langle 1 \right| - \left| 1 \right\rangle \left\langle 1 \right|} \right) = \frac{1}{\sqrt 2 }\left[ {\begin{array}{*{20}c} 1 & 1 \\ 1 & { - 1} \\ \end{array} } \right]$$

In Tsai et al.’s schemes^35–37^, the communicators can randomly decide to perform the unitary operations *I* or *H* on the qubits, and then they measure the qubits using Z-basis, respectively. The relationships between their performed the unitary operations on Bell states and measurement results are calculated in Eqs. (4)–(7) (as shown in Table 1), where *MR*_*A*_ and *MR*_*B*_ represent Alice’s and Bob’s measurement results, respectively, and $\overline{{MR }_{B}}$ denotes the bitwise *NOT* operation on *MR*_*B*_.

4 $$\begin{aligned} & I \otimes I{\mkern 1mu} \left| {\Phi ^{ + } } \right\rangle _{{AB}} = \frac{1}{{\sqrt 2 }}\left( {\left| {00} \right\rangle + \left| {11} \right\rangle } \right)_{{AB}} \\ & I \otimes H{\mkern 1mu} \left| {\Phi ^{ + } } \right\rangle _{{AB}} = \frac{1}{{\sqrt 2 }}\left( {\left| {0 + } \right\rangle + \left| {1 - } \right\rangle } \right)_{{AB}} = \frac{1}{2}\left( {\left| {00} \right\rangle + \left| {01} \right\rangle + \left| {10} \right\rangle - \left| {11} \right\rangle } \right)_{{AB}} \\ & H \otimes I{\mkern 1mu} \left| {\Phi ^{ + } } \right\rangle _{{AB}} = \frac{1}{{\sqrt 2 }}\left( {\left| { + 0} \right\rangle + \left| { - 1} \right\rangle } \right)_{{AB}} = \frac{1}{2}\left( {\left| {00} \right\rangle + \left| {01} \right\rangle + \left| {10} \right\rangle - \left| {11} \right\rangle } \right)_{{AB}} \\ & H \otimes H{\mkern 1mu} \left| {\Phi ^{ + } } \right\rangle _{{AB}} = \frac{1}{{\sqrt 2 }}\left( {\left| { + + } \right\rangle + \left| { - - } \right\rangle } \right)_{{AB}} = \frac{1}{{\sqrt 2 }}\left( {\left| {00} \right\rangle + \left| {11} \right\rangle } \right)_{{AB}} \\ \end{aligned}$$

5 $$\begin{aligned} & I \otimes I{\mkern 1mu} \left| {\Phi ^{ - } } \right\rangle _{{AB}} = \frac{1}{{\sqrt 2 }}\left( {\left| {00} \right\rangle - \left| {11} \right\rangle } \right)_{{AB}} \\ & I \otimes H{\mkern 1mu} \left| {\Phi ^{ - } } \right\rangle _{{AB}} = \frac{1}{{\sqrt 2 }}\left( {\left| {0 + } \right\rangle - \left| {1 - } \right\rangle } \right)_{{AB}} = \frac{1}{2}\left( {\left| {00} \right\rangle + \left| {01} \right\rangle - \left| {10} \right\rangle + \left| {11} \right\rangle } \right)_{{AB}} \\ & H \otimes I{\mkern 1mu} \left| {\Phi ^{ - } } \right\rangle _{{AB}} = \frac{1}{{\sqrt 2 }}\left( {\left| { + 0} \right\rangle - \left| { - 1} \right\rangle } \right)_{{AB}} = \frac{1}{2}\left( {\left| {00} \right\rangle - \left| {01} \right\rangle + \left| {10} \right\rangle + \left| {11} \right\rangle } \right)_{{AB}} \\ & H \otimes H{\mkern 1mu} \left| {\Phi ^{ - } } \right\rangle _{{AB}} = \frac{1}{{\sqrt 2 }}\left( {\left| { + + } \right\rangle - \left| { - - } \right\rangle } \right)_{{AB}} = \frac{1}{{\sqrt 2 }}\left( {\left| {01} \right\rangle + \left| {10} \right\rangle } \right)_{{AB}} \\ \end{aligned}$$

6 $$\begin{aligned} & I \otimes I{\mkern 1mu} \left| {\Psi ^{ + } } \right\rangle _{{AB}} = \frac{1}{{\sqrt 2 }}\left( {\left| {01} \right\rangle + \left| {10} \right\rangle } \right)_{{AB}} \\ & I \otimes H{\mkern 1mu} \left| {\Psi ^{ + } } \right\rangle _{{AB}} = \frac{1}{{\sqrt 2 }}\left( {\left| {0 - } \right\rangle + \left| {1 + } \right\rangle } \right)_{{AB}} = \frac{1}{2}\left( {\left| {00} \right\rangle - \left| {01} \right\rangle + \left| {10} \right\rangle + \left| {11} \right\rangle } \right)_{{AB}} \\ & H \otimes I{\mkern 1mu} \left| {\Psi ^{ + } } \right\rangle _{{AB}} = \frac{1}{{\sqrt 2 }}\left( {\left| { + 1} \right\rangle + \left| { - 0} \right\rangle } \right)_{{AB}} = \frac{1}{2}\left( {\left| {00} \right\rangle + \left| {01} \right\rangle - \left| {10} \right\rangle + \left| {11} \right\rangle } \right)_{{AB}} \\ & H \otimes H{\mkern 1mu} \left| {\Psi ^{ + } } \right\rangle _{{AB}} = \frac{1}{{\sqrt 2 }}\left( {\left| { + - } \right\rangle + \left| { - + } \right\rangle } \right)_{{AB}} = \frac{1}{{\sqrt 2 }}\left( {\left| {00} \right\rangle - \left| {11} \right\rangle } \right)_{{AB}} \\ \end{aligned}$$

7 $$\begin{aligned} & I \otimes I{\mkern 1mu} \left| {\Psi ^{ - } } \right\rangle _{{AB}} = \frac{1}{{\sqrt 2 }}\left( {\left| {01} \right\rangle - \left| {10} \right\rangle } \right)_{{AB}} \\ & I \otimes H{\mkern 1mu} \left| {\Psi ^{ - } } \right\rangle _{{AB}} = \frac{1}{{\sqrt 2 }}\left( {\left| {0 - } \right\rangle - \left| {1 + } \right\rangle } \right)_{{AB}} = \frac{1}{2}\left( {\left| {00} \right\rangle - \left| {01} \right\rangle - \left| {10} \right\rangle + \left| {11} \right\rangle } \right)_{{AB}} \\ & H \otimes I{\mkern 1mu} \left| {\Psi ^{ - } } \right\rangle _{{AB}} = \frac{1}{{\sqrt 2 }}\left( {\left| { + 1} \right\rangle - \left| { - 0} \right\rangle } \right)_{{AB}} = \frac{1}{2}\left( { - \left| {00} \right\rangle + \left| {01} \right\rangle + \left| {10} \right\rangle + \left| {11} \right\rangle } \right)_{{AB}} \\ & H \otimes H{\mkern 1mu} \left| {\Psi ^{ - } } \right\rangle _{{AB}} = \frac{1}{{\sqrt 2 }}\left( {\left| { + - } \right\rangle - \left| { - + } \right\rangle } \right)_{{AB}} = \frac{{ - 1}}{{\sqrt 2 }}\left( {\left| {01} \right\rangle - \left| {10} \right\rangle } \right)_{{AB}} \\ \end{aligned}$$

**Table 1:** Relationship between measurement results and unitary operations.

Initial state	Alice’s operation	Bob’s operation	Quantum state	Relationship of measurement result
$\left| {\Phi^{ + } } \right\rangle_{AB}$	I	I	$\frac{1}{\sqrt 2 }\left( {\left| {00} \right\rangle + \left| {11} \right\rangle } \right)_{AB}$	${MR}_{A}={MR}_{B}$
	I	H	$\frac{1}{2}\left( {\left| {00} \right\rangle + \left| {01} \right\rangle + \left| {10} \right\rangle - \left| {11} \right\rangle } \right)_{AB}$	Uncertain
	H	I	$\frac{1}{2}\left( {\left| {00} \right\rangle + \left| {01} \right\rangle + \left| {10} \right\rangle - \left| {11} \right\rangle } \right)_{AB}$	Uncertain
	H	H	$\frac{1}{\sqrt 2 }\left( {\left| {00} \right\rangle + \left| {11} \right\rangle } \right)_{AB}$	${MR}_{A}={MR}_{B}$
$\left| {\Phi^{ - } } \right\rangle_{AB}$	I	I	$\frac{1}{\sqrt 2 }\left( {\left| {00} \right\rangle - \left| {11} \right\rangle } \right)_{AB}$	${MR}_{A}={MR}_{B}$
	I	H	$\frac{1}{2}\left( {\left| {00} \right\rangle + \left| {01} \right\rangle - \left| {10} \right\rangle + \left| {11} \right\rangle } \right)_{AB}$	Uncertain
	H	I	$\frac{1}{2}\left( {\left| {00} \right\rangle - \left| {01} \right\rangle + \left| {10} \right\rangle + \left| {11} \right\rangle } \right)_{AB}$	Uncertain
	H	H	$\frac{1}{\sqrt 2 }\left( {\left| {01} \right\rangle + \left| {10} \right\rangle } \right)_{AB}$	${MR}_{A}=\overline{{MR }_{B}}$
$\left| {\Psi^{ + } } \right\rangle_{AB}$	I	I	$\frac{1}{\sqrt 2 }\left( {\left| {01} \right\rangle + \left| {10} \right\rangle } \right)_{AB}$	${MR}_{A}=\overline{{MR }_{B}}$
	I	H	$\frac{1}{2}\left( {\left| {00} \right\rangle - \left| {01} \right\rangle + \left| {10} \right\rangle + \left| {11} \right\rangle } \right)_{AB}$	Uncertain
	H	I	$\frac{1}{2}\left( {\left| {00} \right\rangle + \left| {01} \right\rangle - \left| {10} \right\rangle + \left| {11} \right\rangle } \right)_{AB}$	Uncertain
	H	H	$\frac{1}{\sqrt 2 }\left( {\left| {00} \right\rangle - \left| {11} \right\rangle } \right)_{AB}$	${MR}_{A}={MR}_{B}$
$\left| {\Psi^{ - } } \right\rangle_{AB}$	I	I	$\frac{1}{\sqrt 2 }\left( {\left| {01} \right\rangle - \left| {10} \right\rangle } \right)_{AB}$	${MR}_{A}=\overline{{MR }_{B}}$
	I	H	$\frac{1}{2}\left( {\left| {00} \right\rangle - \left| {01} \right\rangle - \left| {10} \right\rangle + \left| {11} \right\rangle } \right)_{AB}$	Uncertain
	H	I	$\frac{1}{2}\left( { - \left| {00} \right\rangle + \left| {01} \right\rangle + \left| {10} \right\rangle + \left| {11} \right\rangle } \right)_{AB}$	Uncertain
	H	H	$\frac{ - 1}{{\sqrt 2 }}\left( {\left| {01} \right\rangle - \left| {10} \right\rangle } \right)_{AB}$	${MR}_{A}=\overline{{MR }_{B}}$

### Encryption chain for the encoding function

Suppose all the four cases (i.e., *I*⊗*I*, *I*⊗*H*, *H*⊗*I*, *H*⊗*H*) are evenly distributed; then only the qubits in *I*⊗*I* and *H*⊗*H* can be used as the secret key bits or checking bits. Based on the relationship mentioned above (see also Table 1), Alice and Bob only have a 50% probability of performing the same unitary operations. Hence, they can use their measurement results as secret key bits or check bits only with 50% of probability.

**Figure 1:**
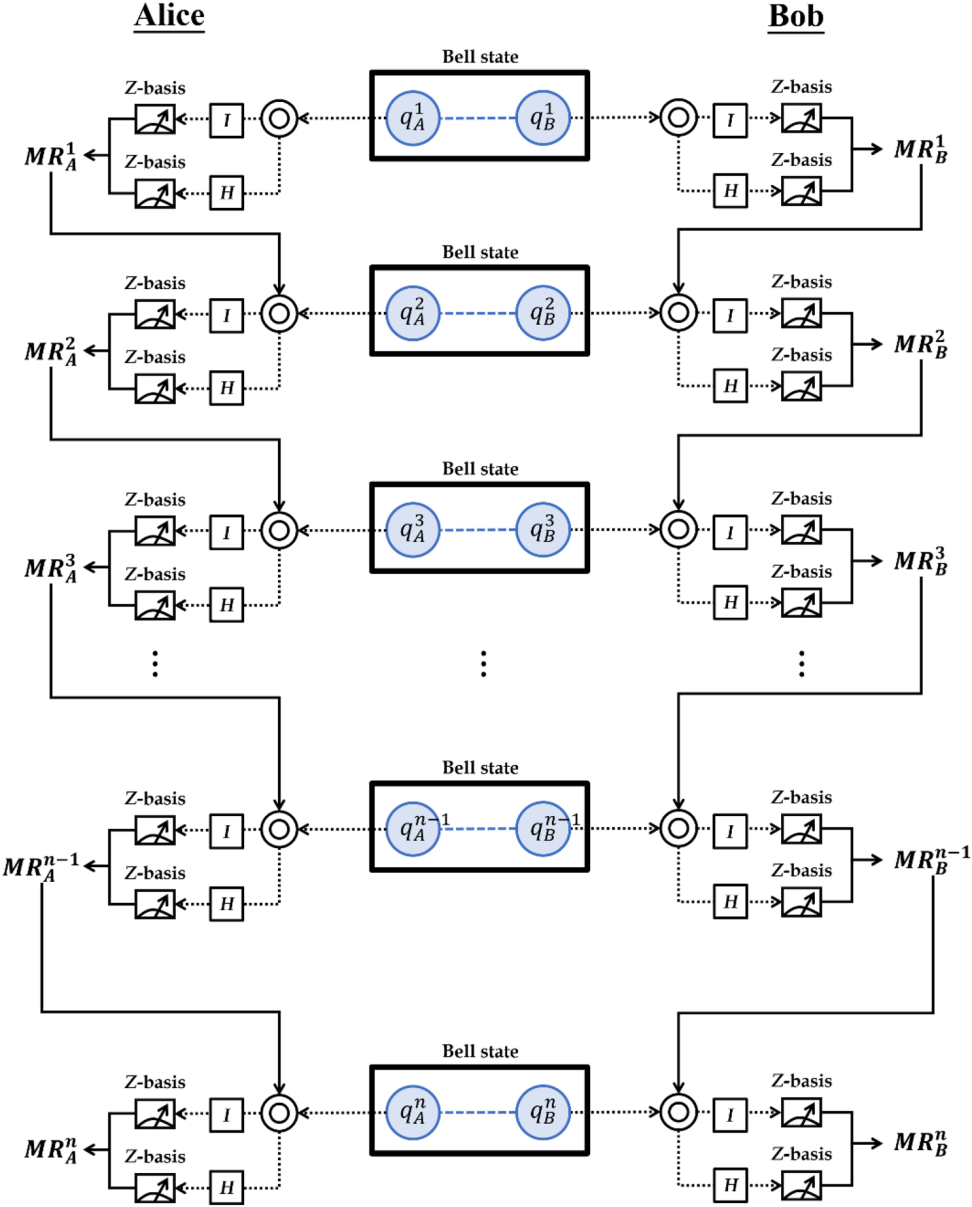
Concept of the coding function.

To improve the qubit efficiency, Alice and Bob decide to perform the unitary operations *I* or *H* on *q*_*A*_ and *q*_*B*_ based on their previous measurement results, ${MR}_{A}^{i}$ and ${MR}_{B}^{i}$, where *i* represents the *i*-th time measurement result. The concept of the coding function is illustrated in Fig. 1. We first prepare the Bell state $\left| {\Phi^{ + } } \right\rangle = \frac{1}{\sqrt 2 }\left( {\left| {00} \right\rangle + \left| {11} \right\rangle } \right)_{AB}$ as the quantum carrier, where $|0\rangle$ represents the classical bit “0” and $|1\rangle$ represents the classical bit “1”. The encoding function is expressed as follows:

- If *i* = 0, then Bob randomly decides to perform the unitary operations *I* or *H* on qubit ${q}_{B}^{i}$ to obtain *q*′${}_{B}^{i}$. Then, he measures qubit *q*′${}_{B}^{i}$ to obtain the measurement result ${MR}_{B}^{i}$ using Z-basis.
- If *i* = 1 ~ *n*, then Bob performs the unitary operations *I* or *H* based on the measurement result ${MR}_{B}^{i-1}=0/1$. For ${MR}_{B}^{i-1}=0$, Bob performs the identity operator *I* on qubit ${q}_{B}^{i}$ to obtain *q*′${}_{B}^{i}$. Then, he measures the qubit *q*′${}_{B}^{i}$ to obtain the measurement result ${MR}_{B}^{i}$ using Z-basis. Otherwise, ${MR}_{B}^{i-1}=1$, and Bob performs the Hadamard operator *H* on qubit ${q}_{B}^{i}$ and measures it.

### Encryption chain for the decoding function

In the proposed encoding function, it is guaranteed that Alice and Bob always use the same unitary operations (i.e., *I*⊗*I* or *H*⊗*H*), then they will obtain the same measurement results. Hence, based on the Z-basis measurement result of the Bell state $\left| {\Phi^{ + } } \right\rangle = \frac{1}{\sqrt 2 }\left( {\left| {00} \right\rangle + \left| {11} \right\rangle } \right)_{AB}$, a decoding table can be constructed (see Table 2). If Alice and Bob perform the same operations (i.e., *I*⊗*I* or *H*⊗*H*) on 1st and 2nd qubits in the Bell state $\left| {\Phi^{ + } } \right\rangle = \frac{1}{\sqrt 2 }\left( {\left| {00} \right\rangle + \left| {11} \right\rangle } \right)_{AB}$ and also perform the Z-basis measurement on 1st and 2nd qubits, then they can obtain the same measurement result (i.e., “00” or “11”). The concept of a decoding function is described below. Alice’s and Bob’s measurement results (i.e., “00” or “11”) can be used to decide their next operation as either *I*⊗*I* or *H*⊗*H*. The decoding function is expressed as follows.

- If *i* = 0, then Bob announces his operation (i.e., *I* or *H*). Subsequently, Alice can perform the same operation on ${q}_{A}^{i}$ and measure it to obtain the measurement result ${MR}_{A}^{i}$ using Z-basis.
- If *i* = 1 ~ *n*, then Alice performs the unitary operations *I* or *H* based on the measurement result ${MR}_{A}^{i-1}=0/1$. For ${MR}_{A}^{i-1}=0$, Alice performs the identity operator *I* on qubit ${q}_{A}^{i}$ to obtain *q*′${}_{A}^{i}$. Subsequently, she measures qubit *q*′${}_{A}^{i}$ to obtain the measurement result ${MR}_{A}^{i}$ by using Z-basis. Otherwise, ${MR}_{A}^{i-1}=1$, and Alice performs Hadamard operator *H* on qubit ${q}_{A}^{i}$ and measures it.

**Table 2:** Encoding and decoding table.

Initial state	Alice’s operation	Bob’s operation	Quantum state	Relationship between the measurement results	Alice’s and Bob’s next operation
$\left| {\Phi^{ + } } \right\rangle_{AB}$	I	I	$\frac{1}{\sqrt 2 }\left( {\left| {00} \right\rangle + \left| {11} \right\rangle } \right)_{AB}$	${MR}_{A}={MR}_{B}=0$	I
				${MR}_{A}={MR}_{B}=1$	H
	H	H	$\frac{1}{\sqrt 2 }\left( {\left| {00} \right\rangle + \left| {11} \right\rangle } \right)_{AB}$	${MR}_{A}={MR}_{B}=0$	I
				${MR}_{A}={MR}_{B}=1$	H

## Proposed unitary-operation-based SQKD protocol

**Figure 2:**
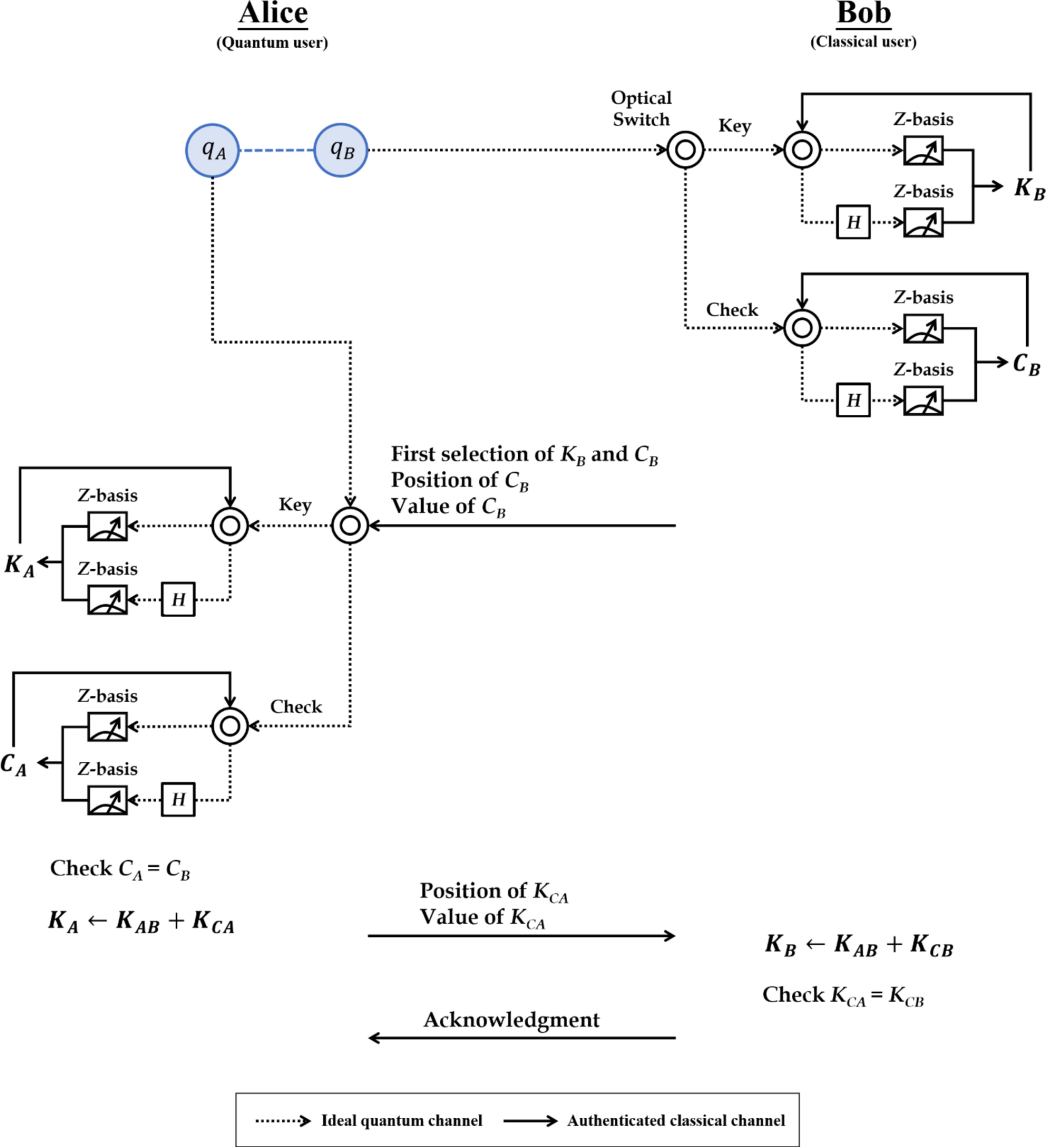
Operational procedure of the proposed SQKD protocol.

In this section, a unitary-operation-based SQKD protocol is presented based on the encryption chain proposed in “Encryption chain based on the measurement result”. Suppose that the quantum channels are ideal and that the classical channels are authenticated. We assume that a quantum communicator (Alice) wants to distribute a secret key with a classical communicator (Bob), which has two quantum capabilities: (1) performing Z-basis measurement and (2) performing identity operator *I* or Hadamard operator *H.* Figure 2 clearly illustrates the proposed unitary-operation-based SQKD protocol. The steps involved in the SQKD protocol are as follows:

Step 1. Alice generates $n$ Bell states in $\left|{\Phi }^{+}\rangle \right.=\frac{1}{\sqrt{2}}(\left|00\rangle \right.+\left|11\rangle \right.)$. She takes the first and second photons from each Bell state to form the order sequences ${S}_{A}=\left\{{q}_{A}^{i}\right\} \mathrm{and} {S}_{B}=\left\{{q}_{B}^{i}\right\}$, for $i=\mathrm{1,2},\dots ,n$. Then, Alice sends ${S}_{B}=\left\{{q}_{B}^{i}\right\}$ to Bob one photon at a time.

Step 2. For every received photon ${q}_{B}^{i}$, Bob randomly selects KEY or CHECK mode. In KEY mode, Bob can perform the following operations:

- If *i* = 0, then Bob randomly decides to perform the unitary operations *I* or *H* on the qubit ${q}_{B}^{i}$ to obtain *q*′${}_{B}^{i}$. Then, he measures the qubit *q*′${}_{B}^{i}$ to obtain the measurement result ${K}_{B}^{i}$ using Z-basis.
- If *i* = 1 ~ *n*, then Bob performs the unitary operations *I* or *H* based on the measurement result ${K}_{B}^{i-1}=0 (1)$. For ${K}_{B}^{i-1}=0 (1)$, Bob performs the identity operator *I* (Hadamard operator *H*) on qubit ${q}_{B}^{i}$ to obtain *q*′${}_{B}^{i}$. Then, he measures the qubit *q*′${}_{B}^{i}$ to obtain the measurement result ${K}_{B}^{i}$ using Z-basis.

In CHECK mode, Bob performs the same operations and records the measurement result ${C}_{B}^{i}$.

Step 3. After Bob completes his operations, he announces the operations of ${K}_{B}^{0}$ and ${C}_{B}^{0}$ (i.e., the operations of the first selection in ${K}_{B}^{i}$ and ${C}_{B}^{i}$), positions of the CHECK mode, and measurement result of ${C}_{B}^{i}$ to Alice via an authenticated classical channel.

Step 4. When Alice receives information from Bob, she can perform the following operations in KEY mode:

- If *i* = 0, then Alice can perform the same operation with Bob on ${q}_{A}^{i}$ and measure it to obtain the measurement result ${K}_{A}^{i}$ using Z-basis.
- If *i* = 1 ~ *n*, then Alice performs the unitary operations *I* or *H* based on the measurement result ${K}_{A}^{i-1}=0 (1)$. For ${K}_{A}^{i-1}=0 (1)$, Alice performs the identity operator *I* (Hadamard operator *H*) on the qubit ${q}_{A}^{i}$ to obtain *q*′${}_{A}^{i}$. Then, she measures qubit *q*′${}_{A}^{i}$ to obtain measurement result ${K}_{A}^{i}$ using Z-basis.

In CHECK mode, Alice performs the same operations and records the measurement result, ${C}_{A}^{i}$.

Step 5. Based on Table 2, Alice can check ${C}_{A}^{i}={C}_{B}^{i}$ for the first eavesdropping check. If eavesdropping is not detected, Alice randomly divides the sequence ${K}_{A}=\left\{{K}_{A}^{i} | i=\mathrm{1,2},\dots ,n\right\}$ into two sequences, namely, ${K}_{AB}$ and ${K}_{CA}$. Further, Alice sends the positions and values of ${K}_{CA}$ to Bob via an authenticated classical channel. Otherwise, Alice asks Bob to abort the process and start a new process.

Step 6. When Bob receives the information from Alice, he can divide the sequence ${K}_{B}=\left\{{K}_{B}^{i} | i=\mathrm{1,2},\dots ,n\right\}$ into two sequences, namely ${K}_{AB}$ and ${K}_{CB}$. Then, he/she can check ${K}_{CA}={K}_{CB}$ for the second eavesdropping check. If eavesdropping is not detected, Bob sends an acknowledgment to Alice and shares the raw key ${K}_{AB}$. Otherwise, Bob asks Alice to abort the process and start a new process. Eventually, if the quantum transmission between Alice and Bob is secure, then they can distil the secret key using the privacy amplification process^69^ on the raw key.

It should be noted that the proposed unitary-operation-based SQKD protocol is secure against Trojan-horse attacks because one-way transmission is adopted. Furthermore, in the proposed SQKD protocol, Alice and Bob can generate the pure-random key because of the property of Z-basis measurements in Bell states. More details of the security and efficiency analyses are provided in “Security analysis” and “Efficiency analysis”, respectively.

## Security analysis

In this section, the security of the proposed SQKD protocol with respect to the three main attacks is discussed.

### Security against collective attack

Collective attacks^70,71^ are a particularly important class of attacks because of their well-known nature such as intercept-and-resend attacks and measure-and-resend attacks. Furthermore, a collective attack is considered as the most general attack^72–75^. Thus, in this study, we prove that the proposed SQKD protocol can be secure against a collective attack to prove the proposed scheme is robust.

Before analyzing the collective attack, we assume an eavesdropper, Eve, who possesses full quantum devices with unlimited computational power and can tamper with the transmitted qubits in the quantum channel. In the collective attack, Eve attempts to eavesdrop on any useful information from Alice and Bob. However, we will prove that Eve cannot reveal any useful information without being detected. In other words, Eve can capture the information, but she will introduce a detectable interruption to the quantum system. Eve performs the collective attack as follows.

In Step 1, Alice sends ${S}_{B}=\left\{{q}_{B}^{i}\right\}$ to Bob one photon at a time. Then, Eve generates ancillary qubits $\left|E\right.\rangle =\left\{\left|{E}_{1}\right.\rangle ,\left|{E}_{2}\right.\rangle ,\dots ,|{E}_{n}\rangle \right\}$ and implements a unitary operation, ${U}_{E}$, on the joint states ${q}_{B}^{i}\otimes \left|{E}_{i}\right.\rangle$. In the proposed SQKD protocol, Alice and Bob perform two eavesdropping checks to verify their measurement result in Steps 5 and 6. To pass the eavesdropping check, Eve considers the following two situations: (1) Alice and Bob perform the same unitary operations $I\otimes I$ and (2) they perform the same unitary operations $H\otimes H$. We assume that Eve performs a unitary operation to attack the transmitted qubit from Alice to Bob in Step 1 using ${U}_{E}$. This can be defined as follows:

8 $$U_{E} \left( {I \otimes I\left| {\Phi^{ + } } \right\rangle \otimes \left| {E_{{\text{i}}} } \right\rangle } \right) = \alpha_{0} \left| {00} \right\rangle \left| {e_{0} } \right\rangle + \alpha_{1} \left| {01} \right\rangle \left| {e_{1} } \right\rangle + \alpha_{2} \left| {10} \right\rangle \left| {e_{2} } \right\rangle + \alpha_{3} \left| {11} \right\rangle \left| {e_{3} } \right\rangle$$

9 $$U_{E} \left( {H \otimes H\left| {\Phi^{ + } } \right\rangle \otimes \left| {E_{{\text{i}}} } \right\rangle } \right) = \frac{1}{2}\left[ {\begin{array}{*{20}c} {\left| {00} \right\rangle \otimes \left( {\alpha_{0} \left| {e_{0} } \right\rangle + \alpha_{1} \left| {e_{1} } \right\rangle + \alpha_{2} \left| {e_{2} } \right\rangle + \alpha_{3} \left| {e_{3} } \right\rangle } \right)} \\ { + \left| {01} \right\rangle \otimes \left( {\alpha_{0} \left| {e_{0} } \right\rangle - \alpha_{1} \left| {e_{1} } \right\rangle + \alpha_{2} \left| {e_{2} } \right\rangle - \alpha_{3} \left| {e_{3} } \right\rangle } \right)} \\ { + \left| {10} \right\rangle \otimes \left( {\alpha_{0} \left| {e_{0} } \right\rangle + \alpha_{1} \left| {e_{1} } \right\rangle - \alpha_{2} \left| {e_{2} } \right\rangle - \alpha_{3} \left| {e_{3} } \right\rangle } \right)} \\ { + \left| {11} \right\rangle \otimes \left( {\alpha_{0} \left| {e_{0} } \right\rangle - \alpha_{1} \left| {e_{1} } \right\rangle - \alpha_{2} \left| {e_{2} } \right\rangle + \alpha_{3} \left| {e_{3} } \right\rangle } \right)} \\ \end{array} } \right]$$

where $\left|{E}_{i}\right.\rangle$ denotes the initial state of Eve’s ancillary qubit; $\left| {e_{0} } \right\rangle$, $\left| {e_{1} } \right\rangle$, $\left| {e_{2} } \right\rangle$, and $\left| {e_{3} } \right\rangle$ are four states that can be distinguished by Eve (i.e., the four states are orthogonal to each other); and $\left| {\alpha_{0} } \right|^{2} + \left| {\alpha_{1} } \right|^{2} + \left| {\alpha_{2} } \right|^{2} + \left| {\alpha_{3} } \right|^{2} = 1$.

In case (1), if Eve passes the eavesdropping check, then she must set $\alpha_{1} = \alpha_{2} = 0$. However, according to this setting, the quantum system for $U_{E} \left( {I \otimes I\left| {\Phi^{ + } } \right\rangle \otimes \left| {E_{{\text{i}}} } \right\rangle } \right)$ can be expressed as follows:

10 $$U_{E} \left( {I \otimes I\left| {\Phi^{ + } } \right\rangle \otimes \left| {E_{{\text{i}}} } \right\rangle } \right) = \alpha_{0} \left| {00} \right\rangle \left| {e_{0} } \right\rangle + \alpha_{3} \left| {11} \right\rangle \left| {e_{3} } \right\rangle$$

In case (2), if Eve passes the eavesdropping check, then she must set $$\alpha_{0} \left| {e_{0} } \right\rangle - \alpha_{1} \left| {e_{1} } \right\rangle + \alpha_{2} \left| {e_{2} } \right\rangle - \alpha_{3} \left| {e_{3} } \right\rangle = \alpha_{0} \left| {e_{0} } \right\rangle + \alpha_{1} \left| {e_{1} } \right\rangle - \alpha_{2} \left| {e_{2} } \right\rangle - \alpha_{3} \left| {e_{3} } \right\rangle = \overset{\lower0.5em\hbox{$\smash{\scriptscriptstyle\rightharpoonup}$}} {\bar{0}}$$. This implies that $$\alpha_{0} \left| {e_{0} } \right\rangle - \alpha_{3} \left| {e_{3} } \right\rangle = \overset{\lower0.5em\hbox{$\smash{\scriptscriptstyle\rightharpoonup}$}} {\bar{0}}$$ signifies $$\alpha_{0} \left| {e_{0} } \right\rangle = \alpha_{3} \left| {e_{3} } \right\rangle$$. However, according to this setting, the quantum system for $$U_{E} \left( {H \otimes H\left| {\Phi^{ + } } \right\rangle \otimes \left| {E_{{\text{i}}} } \right\rangle } \right)$$ can be expressed as follows:

11 $$U_{E} \left( {H \otimes H\left| {\Phi^{ + } } \right\rangle \otimes \left| {E_{{\text{i}}} } \right\rangle } \right) = \frac{1}{2}\left[ {\begin{array}{*{20}c} {\left| {00} \right\rangle \otimes \left( {\alpha_{0} \left| {e_{0} } \right\rangle + \alpha_{3} \left| {e_{3} } \right\rangle } \right)} \\ { + \left| {11} \right\rangle \otimes \left( {\alpha_{0} \left| {e_{0} } \right\rangle + \alpha_{3} \left| {e_{3} } \right\rangle } \right)} \\ \end{array} } \right]$$

In conclusion, if Eve wants to pass the eavesdropping check, then she must make $\alpha_{0} \left| {e_{0} } \right\rangle = \alpha_{3} \left| {e_{3} } \right\rangle$. Eve cannot measure the ancillary qubits $\left|E\right.\rangle =\left\{\left|{E}_{1}\right.\rangle ,\left|{E}_{2}\right.\rangle ,\dots ,|{E}_{n}\rangle \right\}$ to capture the information about Alice’s and Bob’s measurement results because she cannot distinguish $\alpha_{0} \left| {e_{0} } \right\rangle$ from $\alpha_{3} \left| {e_{3} } \right\rangle$. Conversely, if Eve wants to reveal the information about Alice’s and Bob’s measurement results, then she must set $\alpha_{0} \left| {e_{0} } \right\rangle \ne \alpha_{3} \left| {e_{3} } \right\rangle$ (i.e., Eve must make the auxiliary qubit distinguishable). Based on Eq. (11), Eve will disturb the entanglement of the Bell state and will eventually be detected in the eavesdropping check. Therefore, there is no unitary operation for Eve to capture the information about the secret key without being detected. Thus, the proposed SQKD protocol is free from collective attack.

### Security against Trojan horse attack

Trojan horse attacks^64–66^ are common attacks, in which Eve can potentially insert Trojan-horse photons into the transmitted photons sent from Alice. Then, Eve attempts to capture Bob’s information in Step 2 using the measurement result of Trojan-horse photons. However, in the proposed SQKD protocol, the semi-quantum environment (i.e., unitary-operation-based) adopts a one-way transmission strategy as opposed to the round-trip transmission (i.e., randomization-based, measure-resend, and measurement-free). Thus, the classical communicator is not required to be equipped with extra hardware (e.g., photon number splitter and optical wavelength filter devices) to be immune to Trojan-horse attacks.

### Security against intercept-resend attack

**Figure 3:**
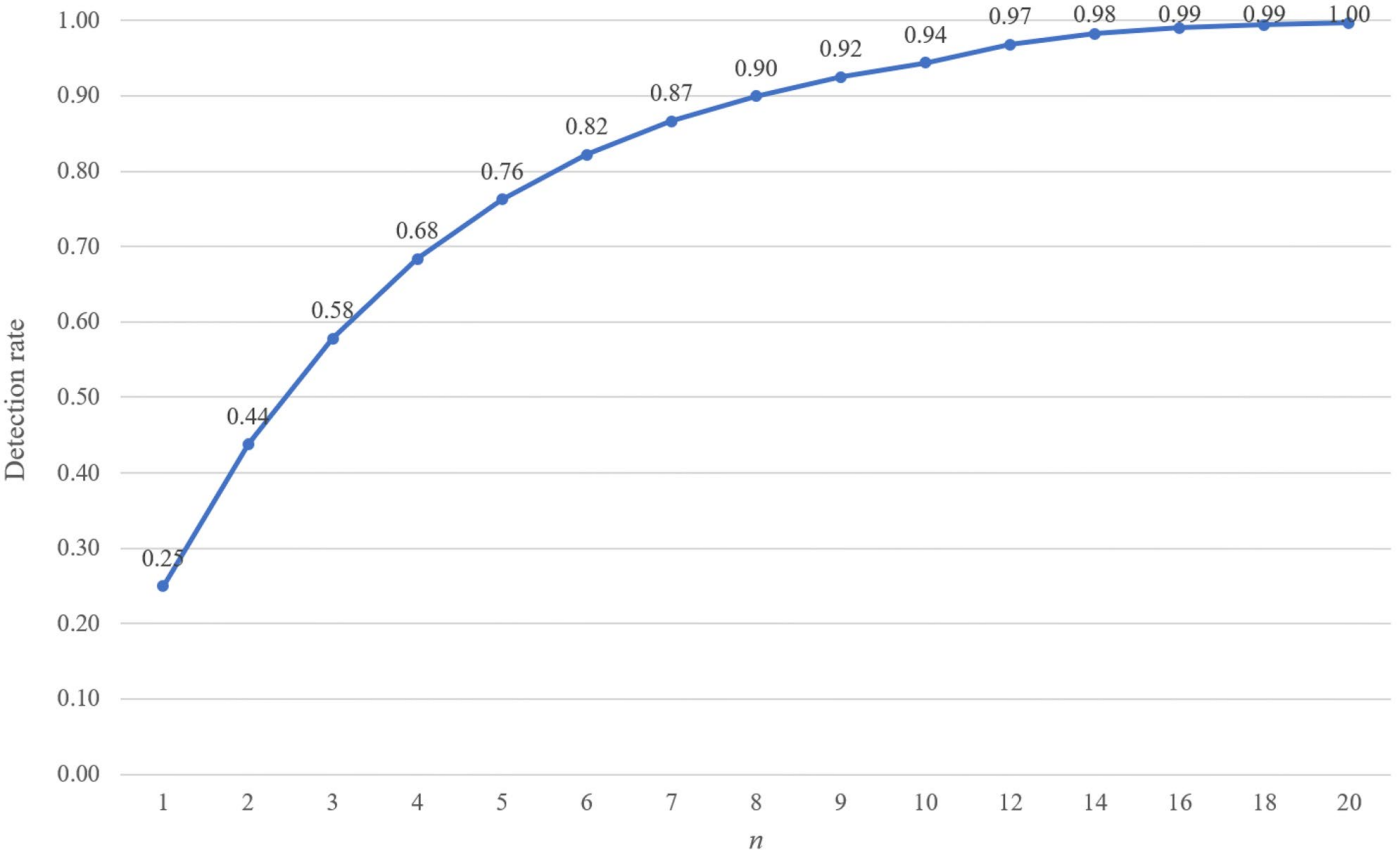
Detection rate of the intercept-resend attack.

In this section, we will analyze the security of the proposed SQKD protocol based on the encryption chain, and we assume the existence of an eavesdropper Eve in the middle of the communication between Alice and Bob, and do a probabilistic security analysis based on the attack pattern that Eve can do. Eve wants to obtain the secret key shared by Alice and Bob, and the attack strategy is based on the principle that the maximum chance of getting the secret key and its existence will not be discovered. Therefore, Eve’s attack mode is to intercept the sequence ${S}_{B}=\left\{{q}_{B}^{i}\right\}$ and guess the unitary operation directly before doing the Z-basis measurement, that is, to do the guessing the unitary operation as identity operator *I* or Hadamard operator *H* for each ${q}_{B}^{i}$ and then do the Z-basis measurement. However, if Eve performs a different unitary operation than the original one, the measurement result will be uncertain, with a 50% chance of being “0” or “1”, i.e., there is a 50% chance of using the wrong unitary operation to measure the correct result. Therefore, by performing the intercept-resend attack, the eavesdropper can pass the eavesdropping check with a probability of ${\left(\frac{3}{4}\right)}^{n}$(assuming that the total number of ${q}_{B}^{i}$ transmitted is *n*). The probability of ${\left(\frac{3}{4}\right)}^{n}$ is the same as that of the BB84 protocol^7^. Thus, the probability to detect the intercept-resend attack in this protocol is ${1-\left(\frac{3}{4}\right)}^{n}$. If *n* is large enough, the detection rate would converge to 1, as shown in Fig. 3.

## Efficiency analysis

Table 3 compares several important parameters of Boyer et al.’s, Wang et al.’s, and Zhou et al.’s SQKD protocols with those of the proposed SQKD protocol. We consider $\eta = \frac{c}{q}$ as the qubit efficiency of a quantum cryptographic protocol^76–78^, where *c* denotes the total number of shared secret bits and *q* denotes the total number of qubits generated by the protocol. Furthermore, we assume that half of the qubits transmitted in the eavesdropping check of the protocol are used to detect the presence of eavesdroppers and the remaining half of the transmitted qubits are used to check for Trojan horse attacks.

**Table 3:** Comparison of various parameters of Boyer et al.’s, Wang et al.’s, and Zhou et al.’s protocols and the proposed protocol.

	Boyer et al.’s protocol^34^	Boyer et al.’s protocol^34^	Wang et al.’s protocol^36^	Wang et al.’s protocol^36^	Zhou et al.’s protocol^46^	Proposed protocol
Semi-quantum environment	Randomization-based	Measure-resend	Randomization-based	Measure-resend	Measure-resend	Unitary-operation-based
Quantum capability of classical participant	(1) Measurement (2) Reorder photons (3) Reflection	(1) Generation (2) Measurement (3) Reflection	(1) Measurement (2) Reorder photons (3) Reflection	(1) Generation (2) Measurement (3) Reflection	(1) Generation (2) Measurement (3) Reflection	(1) Measurement (2) Operation
Quantum resource	Single photon	Single photon	Bell state	Bell state	Cluster state	Bell state
Qubit efficiency	6.25%	6.25%	6.25%	6.25%	3.125%	12.5%
Qubit measurement for Quantum user	Z-basis measurement X-basis measurement	Z-basis measurement X-basis measurement	Bell measurement Z-basis measurement	Bell measurement Z-basis measurement	Z-basis measurement Bell measurement Cluster-basis measurement	Z-basis measurement
Quantum communication	Round-trip	Round-trip	Round-trip	Round-trip	Round-trip	One-way
Vulnerability to Trojan-horse attacks	No	No	No	No	No	No
Photon number splitter	Yes	Yes	Yes	Yes	Yes	No
Wavelength filter	Yes	Yes	Yes	Yes	Yes	No

In Boyer et al.’s SQKD protocols, Alice prepares *n* single photons (i.e., $\left|0\rangle , \left|1\right.\rangle ,\left|+\right.\rangle ,|-\rangle \right.$), and each single photon can be used to share 1-bit raw key. Bob has a 50% chance of choosing the share mode and a 50% chance of choosing the check mode. In share mode, Bob has a 50% chance of using the right basis and a 50% chance of using the wrong basis. Besides, one round of public discussion was used in the share mode, and half of the transmitted qubits were used to check for Trojan horse attacks. Therefore, the qubit efficiency of Boyer et al.’s SQKD protocols corresponded to $\frac{n}{n}\times \frac{1}{2}\times \frac{1}{2}\times \frac{1}{2}\times \frac{1}{2}=\frac{1}{16}=6.25\%$.

In Wang et al.’s SQKD protocols, Alice must generate $n$ Bell states (i.e., $2n$ qubits), and each Bell state can be used to share 1-bit raw key. Two rounds of public discussion were used in Wang et al.’s SQKD protocols, and half of the transmitted qubits were used to check for Trojan horse attacks. Therefore, the qubit efficiency of Wang et al.’s SQKD protocols corresponded to $\frac{n}{2n}\times \frac{1}{2}\times \frac{1}{2}\times \frac{1}{2}=\frac{1}{16}=6.25\%$.

In Zhou et al.’s SQKD protocol, Charlie prepares *n* Cluster states (i.e., $4n$ qubits), and each Cluster state can be used to share 2-bit raw key. Alice and Bob each have a 50% chance of choosing the share mode and a 50% chance of choosing the check mode. Only when Alice and Bob select share mode at the same time, they can use it for sharing the secret key. The chance of this happening is only 25%. Besides, one round of public discussion was used in the share mode, and half of the transmitted qubits were used to check for Trojan horse attacks. Therefore, the qubit efficiency of Zhou et al.’s SQKD protocol corresponded to $\frac{2n}{4n}\times \frac{1}{4}\times \frac{1}{2}\times \frac{1}{2}=\frac{1}{32}=3.125\%$.

In the proposed SQKD protocol, each Bell state can be used to encode 1-bit raw key. Alice generates $n$ Bell states ($2n$ qubits). Two rounds of public discussion are conducted in the proposed SQKD protocol. Therefore, the qubit efficiency of the proposed SQKD protocol is $\frac{n}{2n}\times \frac{1}{2}\times \frac{1}{2}=\frac{1}{8}=12.5\%$. Obviously, the qubit efficiency of the proposed SQKD protocol is twice that of Boyer et al.’s and Wang et al.’s SQKD protocols. The qubit efficiency of the proposed SQKD protocol is four times higher than that of Zhou et al.’s SQKD protocol. The SQKD protocols proposed by Wang et al., Boyer et al., and Zhou et al. are vulnerable to Trojan horse attacks. Furthermore, the qubit efficiency of Wang et al.’s, Boyer et al.’s, and Zhou et al.’s SQKD protocols decrease to 50% if a photon number splitter and wavelength filter are applied to avoid Trojan horse attacks. Moreover, in Wang et al.’s SQKD protocols, the quantum user (Alice) must perform Bell-basis and Z-basis measurements because of the design of the eavesdropping check. In Zhou et al.’s SQKD protocol, the quantum user (Alice) must perform Cluster-basis, Bell-basis, and Z-basis measurements because of the design of the eavesdropping check. Therefore, in the proposed SQKD protocol, Alice is required to solely implement the measurement of single photons, which is simpler than Cluster-basis and Bell-basis measurements.

## Conclusion

In this study, a new coding function, also known as an encryption chain based on the measurement result, was proposed. A novel unitary-operation-based SQKD protocol was designed based on this new coding function. The proposed SQKD protocol is more efficient and practical than the existing SQKD protocols because it is designed based on one-way transmission as opposed to round-trip transmission, which is congenitally immune to Trojan horse attacks without the need of any extra hardware. Moreover, security analysis showed that the proposed SQKD protocol can avoid collective attacks. Additionally, the proposed SQKD protocol provides the best qubit efficiency among the existing SQKD protocols, and classical participants are required to possess only two quantum capabilities, which enhances its practicability. Furthermore, the proposed coding function can be useful in applications involving semi-quantum secret sharing protocols and semi-quantum communication protocols for improving qubit efficiency. However, this requires further investigation.

## Data Availability

All data generated or analysed during this study are included in this published article.
